# Empathy promotes altruistic behavior in economic interactions

**DOI:** 10.1038/srep31961

**Published:** 2016-08-31

**Authors:** Olga M. Klimecki, Sarah V. Mayer, Aiste Jusyte, Jonathan Scheeff, Michael Schönenberg

**Affiliations:** 1 Swiss Centre for Affective Sciences, University of Geneva, Geneva, Switzerland; 2 Department of Clinical Psychology and Psychotherapy, University of Tübingen, Tübingen, Germany; 3 LEAD Graduate School & Research Network, University of Tübingen, Tübingen, Germany

## Abstract

What are the determinants of altruism? While economists assume that altruism is mainly driven by fairness norms, social psychologists consider empathy to be a key motivator for altruistic behavior. To unite these two theories, we conducted an experiment in which we compared behavior in a standard economic game that assesses altruism (the so-called Dictator Game) with a Dictator Game in which participants’ behavioral choices were preceded either by an empathy induction or by a control condition without empathy induction. The results of this within-subject manipulation show that the empathy induction substantially increased altruistic behavior. Moreover, the increase in experienced empathy predicted over 40% of the increase in sharing behavior. These data extend standard economic theories that altruism is based on fairness considerations, by showing that empathic feelings can be a key motivator for altruistic behavior in economic interactions.

In today’s society, we are faced with numerous global challenges, ranging from the spread of dangerous diseases to a major refugee crisis unparalleled in magnitude since World War II^1^. If we are to meet these challenges successfully, it is crucial that people engage in altruistic acts such as providing medical support or integrating refugees. Although altruism is defined as a costly act performed for the benefit of another^2^, humans and other animals do indeed exhibit and act upon this genuine concern for another’s welfare^3^. Decades of research in the fields of economics^2^,^4^ and psychology^5^ have been dedicated to elucidating the determinants of altruistic behavior. However, previous work on altruistic behavior in these two disciplines has largely been carried out in isolation.

In economics, the long held assumption of the homo economicus positing that individuals strive to maximize their gains was abandoned in the 1980s. Empirical evidence has demonstrated that participants behave more generously than predicted by rational choice theory^6^,^7^. A substantial part of this work employed fairly simple experimental paradigms, particularly the Dictator Game (DG). In the DG, a participant (the ‘dictator’) can freely divide a given amount of money between himself and an anonymous recipient, who has no choice but to accept the monetary distribution^8^. While the assumption of the homo economicus predicts that participants will not share any money with the recipient, a meta-analysis taking into account hundreds of studies using the DG has shown that on average, participants give about 30% of their endowment^4^. As this behavior is far more generous than expected, experimental economists have extensively investigated the determinants underlying altruistic behavior. Evidence from these studies has provided the basis for the widely accepted idea that social norms related to fairness (i.e., striving for equal material benefits for oneself and others) play a key role in altruism^9^,^10^.

However, the psychological factors that drive this kind of altruistic behavior have been poorly understood. This is surprising considering that psychological research^5^,^11^,^12^ has established a close link between altruism and emotional empathy, the capacity to share the feelings of another^3^,^12^. In fact, the connection between altruism and empathic feelings for others in need was proposed as early as the 18^th^ century by Adam Smith when he wrote “How selfish soever man may be supposed, there are evidently some principles in his nature, which interest him in the fortune of others, and render their happiness necessary to him, though he derives nothing from it, except the pleasure of seeing it. Of this kind is pity or compassion, the emotion we feel for the misery of others” (ref. 13, *page 3*). Meta-analytic evidence from psychology corroborates this historical postulate by revealing that empathic states and, to a smaller extent, empathic traits predict altruistic behavior^5^. In line with these findings, the empathy-altruism hypothesis^11^ has posited that altruistic motivation is elicited by empathy felt for a person in need. More recently, researchers have suggested that in both humans and animals empathy has evolved in order to promote altruism towards others in need, pain, or distress^3^.

Surprisingly, despite these developments in psychology and economics, there have been few attempts to integrate the knowledge from both fields in order to further the understanding of altruism. So far it has been established that empathic traits are positively related to donations in the standard DG^14^. Previous research also showed that compassion, which denotes a feeling of care for a suffering other accompanied by the desire to help^15^, motivates prosocial behavior in less religious individuals^16^. In spite of these first attempts to bridge the psychological concept of empathy (or the related concept of compassion) with economic measures of prosociality, to our knowledge no previous study has tested whether the degree of empathy experienced for the recipient represents the key mechanism motivating prosocial behavior towards this target^3^,^5^,^11^ in economic contexts. To address this question, the current study examined whether a within-subject manipulation of empathic states can increase altruistic behavior towards specific others in the DG and whether inter-individual differences in empathic experiences can explain altruistic behavior in this economic context.

## Methods

**Figure 1:**
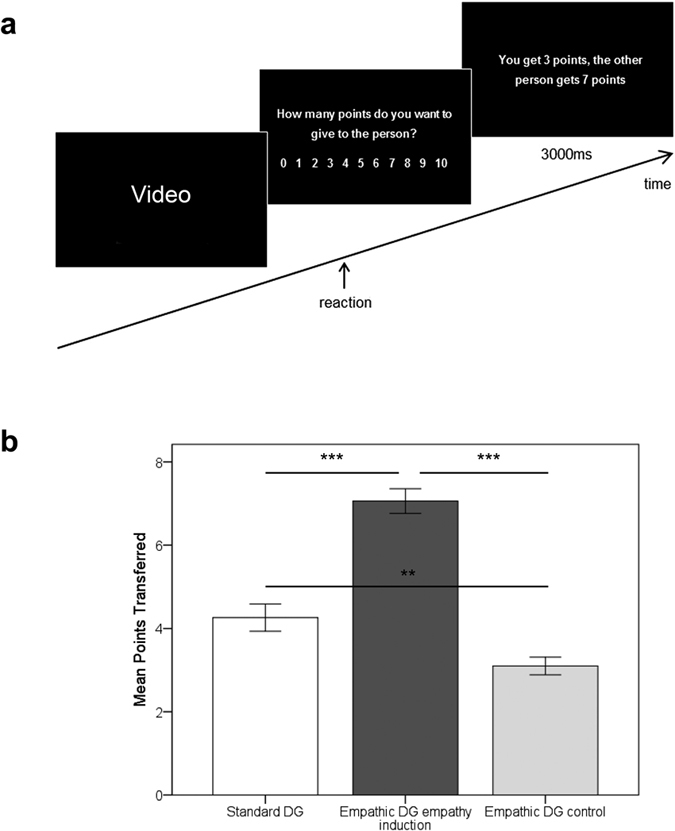
Empathy-induction increased altruistic behavior. (**a**) Timeline of the Empathic DG. After seeing suffering others (empathy induction) or others in everyday activities (control condition), participants indicated by button press how many points they wanted to share with the person(s) in the video. A subsequent screen presented the results of the decision. (**b**) Participants (*n* = 50) transferred more points following the empathy induction as opposed to the control condition and the standard DG. Asterisks denote levels of statistical significance of pairwise comparisons with ***p < 0.001 and **p < 0.01; error bars denote s.e.m.

In order to test whether the empathy-altruism hypothesis holds in the realm of economic interactions, we conducted a within-subject experiment in which we compared the standard one-shot DG with a novel version of the DG, the so-called Empathic DG (Fig. 1a). In the Empathic DG, economic choices are either preceded by an empathy induction or by a control condition. The standard one-shot DG, with a recipient who is randomly chosen from a pool of other players, was included to enable a comparison with previous research. Both versions of the DG (standard and Empathic) were played with real monetary stakes (10 monetary units, MUs, per trial). A total of 50 participants (mean age = 23.72 years; 31 females) first played the standard DG, followed by the Empathic DG. In the Empathic DG, empathy was elicited through the presentation of videos depicting a suffering person in need (e.g., a helpless child in an orphanage). This empathy induction was inspired by previous work^17^ showing that i) empathy can be induced when participants are confronted with suffering and that ii) the elicited empathic feelings vary between participants. In the control condition of the Empathic DG, participants viewed videos of people performing everyday activities (e.g., two individuals talking to each other). All the videos were selected from the Socio-affective Video Task^18^. Gender, race, and age were balanced across empathy-inducing and control videos. This was confirmed by Pearson’s chi-square tests that revealed no significant difference for gender (*χ^2^*(2) = 2, *p* = 0.42), age (*χ^2^*(2) = 3.3, *p* = 0.22), or race (*χ^2^*(1) = 1.5, *p* = 0.36). After each of the 44 randomly presented documentary videos (half of them inducing empathy, the other half being control videos), participants determined how many MUs they gave to the person(s) in the video. Following the Empathic DG, participants watched each video again and provided self-reported ratings of positive affect, negative affect, and empathy (on scales ranging from 0, not at all, to 10, very strong). More specifically, we asked them to answer the following questions: “To what degree did you experience positive emotions during the video?”, “To what degree did you experience negative emotions during the video?”, and “How much empathy did you feel?”. Trait empathy was assessed prior to the study using the Interpersonal Reactivity Index (IRI)^19^. In line with the definition of fairness as the striving for equal benefits for oneself and others^9^,^10^, an even split of MUs between the participant and the recipient was used as an indicator of fairness considerations. All participants provided written informed consent and received monetary compensation for participation. The study protocol was approved by the ethics committee of the University of Tübingen and was carried out in accordance with the approved guidelines and the declaration of Helsinki.

## Results

### Manipulation check

To check whether empathy-inducing videos elicited different affective states than control videos in the current sample, we first conducted a repeated measures multivariate analysis of variance (MANOVA) with the within subject variable video-type (empathy-inducing vs control videos) and the dependent variables empathy, positive affect, and negative affect. This analysis revealed a significant effect of video-type (*F*(3,47) *=* 236.31, *p* < 0.001, *η*^*2*^ = 0.94). Follow-up pairwise comparisons confirmed that empathy-inducing videos indeed elicited more empathy and negative feelings than control videos, while control videos elicited more positive feelings than empathy-inducing videos (all *p* < 0.001; for details, see Supplementary Fig. S1).

### Giving behavior

Using a repeated measures ANOVA, we assessed the effect of the empathy induction on altruistic behavior by comparing the amount of MUs shared with the recipient in the standard DG with the amount of MUs shared following the empathy induction or the control condition in the Empathic DG. In line with our assumptions, we observed a large effect of condition (*F*(2,48) = 155.35, *p* < 0.001, *η*^*2*^ = 0.87), which was significant for all pairwise comparisons (see Fig. 1b for details) and showed that the empathy induction increased helping behavior. Following the empathy induction, participants gave 70.59% of their total endowment, whereas they only gave 42.60% in the standard DG and 30.99% in the control condition.

### Regression Analyses

**Figure 2:**
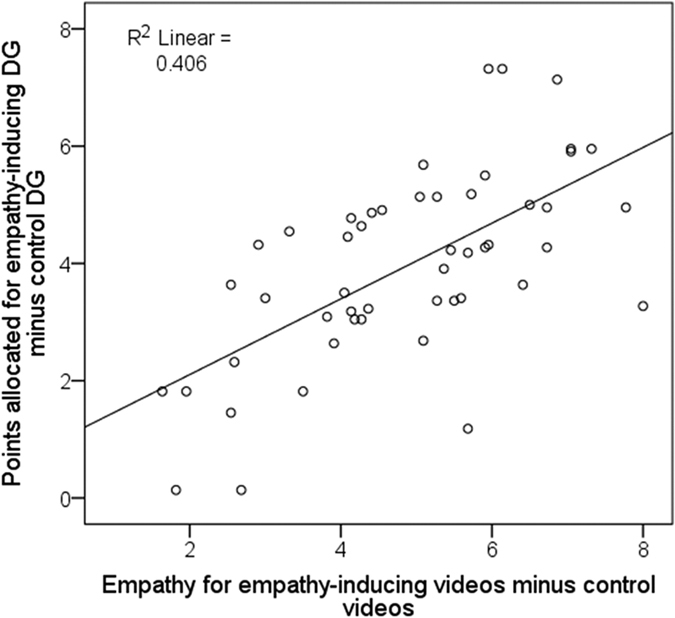
Mean increase of self-reported feelings of empathy from control to empathy-inducing videos predicted mean increase in altruistic behavior from control to empathy-inducing videos (*n* = 50) in the Empathic DG.

Regression analyses were computed to determine which state components predicted giving behavior. A first regression analysis revealed that empathy significantly predicted generosity in response to empathy-inducing videos (*b* = 0.75, *p* < 0.001), explaining 31.2% of the variance (*R*^2^ = 0.31, *F*(1,48) = 21.73, *p* < 0.001; Fig. 2). Negative emotions also predicted generosity in response to empathy-inducing videos (*b* = 0.46, *p* < 0.01), explaining 19% of the variance (*R*^2^ = 0.19, *F*(1,48) = 11.23, *p* < 0.01). A multiple regression analysis that simultaneously included both empathy and negative affect revealed that the effect of empathy remained significant (*b* = 0.65, *p* < 0.01), while the negative affect ratings did not significantly contribute to explaining the variance in this model (*b* = 0.11, *p* = 0.51).

Subsequently, the effect of the empathy induction on altruistic behavior was examined. To this end, we computed a regression analysis that tested whether the increase in empathy from control videos to empathy-inducing videos predicted the increase in the number of MUs allocated from the control to the empathy-inducing condition. The effect of empathy induction on altruistic behavior was significant (*b* = 0.65, *p* < 0.001), explaining 40.6% of the variance (*R*^2^ = 0.41, *F*(1,48) = 32.79, *p* < 0.001; Fig. 2).

Finally, three regression analyses were run to test whether trait empathy (as indicated by the IRI score) predicted altruistic behavior in i) the standard DG, ii) the Empathic DG preceded by an empathy induction and iii) the Empathic DG preceded by a control condition. None of the regressions were significant (all *p* ≥ 0.36).

## Discussion

The present data show that the empathy-altruism hypothesis^11^,^20^ can explain behavior in economic interactions. In line with the assumption of fairness norms, behavior in the standard DG and the control condition of the Empathic DG conformed to the prediction of equitable monetary distributions. However, following the empathy induction, participants were willing to give over 70% of their endowments to the suffering others and this increase was explained by an increase in empathic feelings. In other words, it was possible to overcome fairness norms with an empathy induction specifically linked to the suffering recipient. This finding extends the notion that situational empathy is a central motivator of altruism directed at helping others in need, pain, or distress^3^ to economic interactions. In contrast to previous work, which observed much lower levels of altruism with charities^21^ or fictitious characters^22^ as recipients, the present study reveals that altruism substantially increases when individuals divide real monetary stakes and when they are presented with visual displays of suffering recipients. The current work showed that the degree of empathy experienced towards a specific person changes as a function of the situation and motivates altruistic behavior in an economic context. This finding extends previous evidence on the relation between trait empathy and altruism towards anonymous strangers^14^ as well as the research on the role of compassion as a motivator for prosocial behavior in less religious participants^16^. Moreover, our findings provide an explanation for the observation that putting a face to the victims increases altruistic behavior^23^ by showing that the extent of empathy experienced towards another person strongly predicts how much people are willing to share in economic interactions.

In the present study, self-reports of empathic feelings predicted a large degree of altruistic behavior, whereas empathic traits were not predictive of altruistic behavior. Although this finding contrasts with previous work, which has reported a significant relationship between trait empathy and behavior in a standard DG^14^, it is in line with meta-analytic evidence that prosocial behavior is more strongly related to situational empathy than to empathic traits^5^. These lower correlations for empathic traits might be explained by the heterogeneity of questionnaire measures^5^.

Taken together, the present findings indicate that in order to promote altruism - whether it is for charities, refugees, or in other economic and political contexts - it is essential to appeal to a person’s empathy for specific recipients.

## Additional Information

**How to cite this article**: Klimecki, O. M. *et al*. Empathy promotes altruistic behavior in economic interactions. *Sci. Rep.* **6**, 31961; doi: 10.1038/srep31961 (2016).

## Supplementary Material

Supplementary Information

Supplementary Dataset
