# Supplementary material for: Empathy promotes altruistic behavior in economic interactions
Source: Sci Rep. 2016 Aug 31;6:31961. doi: 10.1038/srep31961 (PMC5005993; doi:10.1038/srep31961)
Supplement: Supplementary Information [file srep31961-s1.pdf]

Supplementary Information

Empathy promotes altruistic behavior in economic interactions

Olga M. Klimecki[1*†], Sarah V. Mayer[2], Aiste Jusyte[3], Jonathan Scheeff[2] and Michael Schönenberg[2]

[1] Swiss Centre for Affective Sciences, University of Geneva, Geneva, Switzerland

[2] Department of Clinical Psychology and Psychotherapy, University of Tübingen, Tübingen, Germany

[3] LEAD Graduate School & Research Network, University of Tübingen, Tübingen, Germany

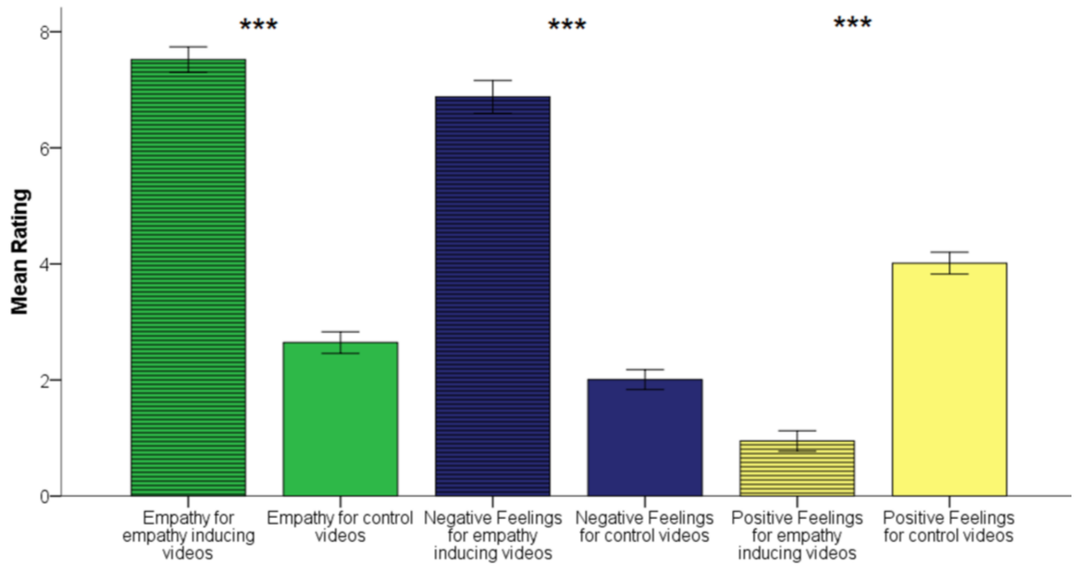

Figure S1. Mean Ratings of Empathy, Negative Feelings and Positive Feelings differed for empathy-inducing and control videos (n = 50). Error bars depict s.e.m., asterisks denote levels of statistical significance of pairwise comparisons with *** p < .001.
